# Podcasts as a tool for promoting health-related behaviours: A scoping review

**DOI:** 10.1177/20552076241288630

**Published:** 2024-10-13

**Authors:** Bethany Robins, Tessa Delaney, Carol Maher, Ben Singh

**Affiliations:** 1 Alliance for Research in Exercise Nutrition and Activity (ARENA), 1067 University of South Australia, Adelaide, SA, Australia; 2 School of Medicine and Public Health, College of Health, Medicine and Wellbeing, 5982 University of Newcastle, Callaghan, NSW, Australia; 3 Hunter Medical Research Institute (HMRI), New Lambton, NSW, Australia; 4 585488 Hunter New England Population Health, Hunter New England Local Health District, Wallsend, NSW, Australia

**Keywords:** Health promotion, media, medical information, podcasts, public health, scoping review

## Abstract

**Background:**

Podcasts are a popular medium for delivering health-related content, potentially influencing physical and mental health behaviours and outcomes. This scoping review evaluates the impact of health podcasts on such outcomes, engagement levels, and public perceptions.

**Methods:**

This scoping review followed the Arksey and O'Malley framework and PRISMA-ScR guidelines. Qualitative or quantitative articles published from 2004 onward were included. Eligibility criteria were developed using the Population, Exposure and Outcome framework. Included articles examined the use, engagement with, or perspectives towards health-related podcasts. Data were synthesised narratively.

**Results:**

Fifty articles encompassing 38 studies were analysed. Significant improvements (*p* < 0.05) were observed in health monitoring, knowledge, behaviours, attitudes, chronic disease management, maternal health and behavioural improvements. Results were mixed for physical activity (*n* = 2 significantly improved, *n* = 2 no change), fruit and vegetable consumption (*n* = 1 significantly improved, *n* = 1 no change), and weight outcomes (*n* = 2 significantly improved, *n* = 2 no change). No significant changes were observed in depression and anxiety. Podcast engagement studies (*n* = 7) reported participation rates between 62% and 83% and an average weekly listening duration of 103–124 minutes. There was high satisfaction, trust and appreciation for podcasts that effectively blended personal anecdotes with reliable medical information. Sample sizes ranged from 7 to 722, with sample age ranging from under 18 to 73.2 ± 6.2 years. Studies included both male (*n* = 1), female (*n* = 6) and mixed samples (*n* = 24). Podcasts were used individually (*n* = 19), alongside other supportive technology such as apps and online material (*n* = 11) and retrospective podcast use was analysed in cross-sectional (*n* = 3) and a single (*n* = 1) audit. Duration of podcasts ranged from 30 seconds to 24 minutes.

**Conclusion:**

Podcasts show promise as effective tools for health promotion, achieving strong engagement and effects on knowledge and behaviours. Future research should explore content innovation and integration into health interventions, and long-term effectiveness.

## Introduction

Podcasts have become immensely popular, offering a versatile platform that covers a wide range of diverse topics such as news, entertainment, sports, health and more. The global podcast landscape has witnessed remarkable growth, with around 465 million listeners in 2023^1^ This surge is expected to continue, with a projected audience of 505 million listeners in 2024.^2^ Among adults who listened to podcasts in 2023, the average listening time was 9.0 hours per week^3^ This expanding reach, combined with the diverse content, highlights the potential of podcasts in promoting health behaviours and impacting wellbeing.^4,5^

Health and wellbeing podcasts can convey a broad spectrum of health information, encompassing topics like medical education, nutrition, fitness and more.^6–11^ Podcasts can simplify complex health topics, making information more relatable and accessible through online and downloadable formats.^12^ A key advantage includes convenience, with users able to listen at any time and location, including while undertaking other daily activities.^12,13^ The diversity of health-related podcasts increases their appeal, providing listeners with a variety of content to inform their health decisions. This accessibility and convenience make them valuable tools for disseminating health information, during day-to-day life. However, research in the area is currently limited.

Podcasts have gained significant attention as educational tools in various fields, prompting several scoping reviews to assess their effectiveness and impact. In medical education, an early scoping review revealed a growing trend in podcast usage, particularly for medical resident training and integration into formal curricula.^9^ This review highlighted learners’ appreciation for podcasts due to their portability, efficiency, and ability to combine education with entertainment. The effectiveness of podcasts was found to be comparable to traditional methods in improving medical students’ documentation skills and prompting practice changes among medical residents and physicians.^9^ Similarly, a scoping review focused on kinesiology education suggested that podcasts could serve as effective learning tools for both practitioners and undergraduate kinesiology students.^10^ However, the authors noted a lack of sufficient detail in research design, instrument development, and findings within the identified studies. This led to their conclusion that more rigorous future research is necessary to fully comprehend the impact of educational podcasts in the field of kinesiology.^10^

The application of podcasts in education extends beyond these disciplines. Additional reviews have explored their use and effectiveness in various contexts, including teaching and learning in occupational therapy,^14^ education of health professionals,^15^ and learning in higher education.^16^ These reviews collectively demonstrate the widespread interest in podcasts as educational tools across multiple disciplines.

Previous scoping reviews have highlighted the growing use of podcasts in medical and kinesiology education, noting their effectiveness and need for more rigorous research. However, there is a lack of reviews on health podcasts aimed at a general audience, indicating a gap in understanding their impact on health-related behaviours. To our knowledge, there has not been a review of health podcasts aimed at a general audience. This indicates a notable gap in the current understanding of the effectiveness and potential impact of podcasts in fostering health-related behaviours. Therefore, this scoping review aims to assess the use and effects of podcasting on health-related behaviours and outcomes in the general population. Specifically, the aims are to:

1. To describe the effects of health-related podcasts on behaviours and outcomes within the general population.
2. Investigate patterns of use and engagement with health-related behaviour podcasts, examining factors such as usage trends, content, length, structure, and barriers to uptake.
3. Explore perspectives towards health podcasts to gain insights into how individuals perceive and interact with this medium.

The insights gained can guide future research directions, inform practice and policy, and contribute to the development of more effective interventions that may use podcasts in healthcare and public health.

## Methods

The protocol for this scoping review was prospectively registered on Open Science Framework (registration ID: https://osf.io/mkz76/?view_only=124487c6035d4a228c2dcc46a8c3ed46). This scoping review adhered to the Preferred Reporting Items for Systematic Reviews and Analyses Extension for Scoping Reviews (PRISMA-ScR) guidelines.^17^ Additionally, the 5-step framework for scoping reviews proposed by Arksey and O'Malley (2005) was followed: (1) identifying the research question, (2) searching for relevant studies, (3) selecting studies, (4) charting the data and (5) collating, summarising and reporting the results.^18^ This framework guided the development of inclusion and exclusion criteria, the systematic search strategy, the screening process, data extraction and presentation of findings.

### Eligibility criteria

All original qualitative and quantitative studies published in peer-reviewed journal articles from 2004 onwards were considered eligible for inclusion. The study eligibility criteria aligned with the Population, Exposure, and Outcome (PEO) framework.^19^ A summary of inclusion and exclusion criteria are provided in Table 1. *Population:* Studies involving participants of any age were included. *Exposure:* Studies that examined the use, engagement with, or perspectives towards health-related podcasts where included. Eligible studies also encompassed evaluations of podcasts’ effects on health outcomes or health-related behaviours. For this review, a ‘podcast’ was defined as an internet-based audio file that can be streamed or downloaded onto a computer or mobile device, typically available as a series of episodes.^20^ Only studies referring to their subject matter explicitly as ‘podcasts’ were considered. Health-related behaviours included any actions affecting health or mortality, such as efforts to improve sleep, activity levels, dietary habits, mental health and overall well-being.^21,22^ A ‘health podcast’ was defined as digital audio content aimed at influencing health behaviours or outcomes. *Outcomes:* Studies were eligible if they evaluated at least one of the following outcomes: (i) use and engagement with health podcasts; (ii) listener perspectives; (iii) educational impacts on the audience; or (iv) the effect of podcasts on health behaviours and outcomes.

**Table 1.** A summary of inclusion and exclusion criteria.

	Included	Excluded
Participants	The general population, regardless of age or health status.	Medical health professionals.
Exposure	Studies that involve evaluation of podcasts, defined as an internet-based audio file which can be streamed or downloaded to a computer or mobile device, typically available as a series of episodes. Must use the term ‘podcast’ to describe the exposure. Podcast subject must focus on health-related behaviours or outcomes and can include narrative pieces (e.g. individuals lived experiences).	Podcasts specifically targeting the education of health and medical professionals, such as doctors, nurses, therapists.
Outcomes	Health-related behaviours and health-related outcomes, including, but not limited to, physical activity, diet, quality of life, sleep, mental health and wellbeing. Podcast usage including usage over time, content, length, uptake and barriers. Attitudes towards podcasts Educational benefits of listening to podcasts including, reaction, learning, behavioural change and results.	Medication adherence.
Study design	Audits of podcasts. Original qualitative and quantitative research.	Dissertations, review articles, conference abstracts, commentary articles and all grey literature. Studies without full text available.

### Search strategy

The following databases were searched: CINAHL, Cochrane, Embase, MEDLINE, Emcare, ProQuest Health and Medical Complete, ProQuest Nursing and Allied Health Source, PsycINFO, Scopus, Sport Discus, EBSCOhost, Web of Science and ERIC. Searches were performed using predefined search terms for ‘podcasts’ and ‘health-related behaviours’ or outcomes (see Supplementary Content 1 for the full search strategy) for peer-reviewed journal articles published in English from inception to 16 September 2023. Additionally, the reference lists of relevant systematic reviews were screened for potentially pertinent articles.

### Selection process

Search results were imported into EndNote x9 (Clarivate, Philadelphia, PA), duplicates were removed, and articles were then exported into Covidence (Veritas Health Innovation, Melbourne, Australia). An independent researcher screened articles based on title and abstract, following by full-text screening. All stages of screening, data extraction and risk of bias scoring were conducted by one reviewer, with 20% being conducted by a second reviewer for accuracy.

### Data management and extraction

Standardised data extraction forms were used to extract the following information from eligible studies: population characteristics, sample size, age, gender, study country, study design, podcast design, purpose of listening, outcome measures and study results.

### Risk of bias assessment

The Mixed Methods Appraisal Tool (MMAT) version 2018^23^ was used to assess bias risk in the included studies. The MMAT is a tool designed for use in reviews involving a mix of quantitative, qualitative, and mixed methods studies. The MMAT contains two screening questions to assess relevance to the review topic, followed by five categories with methodological criteria tailored to appraise the quality of quantitative (randomised controlled, non-randomised and descriptive studies), qualitative and mixed methods studies.

### Synthesis methods

A PRISMA flowchart was used to visually depict the process of identifying and selecting studies for inclusion at each stage of the review, detailing the number of records identified, included, and excluded, and providing reasons for exclusions. The studies were summarised in a study characteristics table, which included comprehensive details on each study's design, purpose, podcast topic, and outcomes, providing a clear overview of the key aspects of the included research. Results pertaining to each study aim were then analysed, ensuring transparency and replicability of the research methodology.

## Results

### Literature search

**Figure 1.**
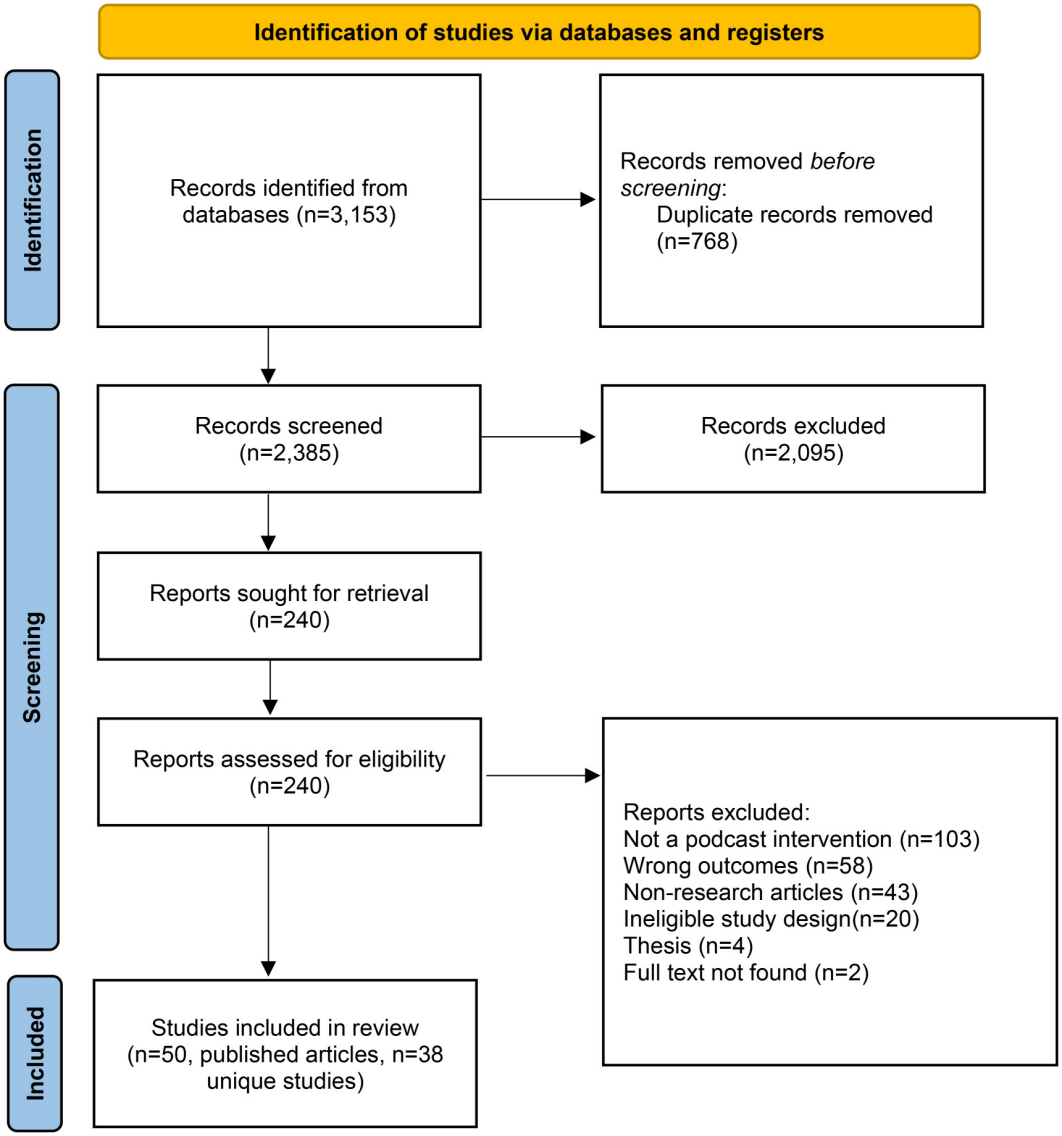
PRISMA flow diagram.

The database searches identified 3153 records (see Figure 1 for PRISMA flow diagram). After removing 768 duplicate articles, 2385 titles and abstracts were screened. Of these, 2095 were excluded. The remaining 290 full-text articles were assessed, and 240 were further excluded. Ultimately, 50 published articles, which reported on 38 unique studies, were included. Sample sizes ranged from 12 to 722 participants, totalling 5094 participants across all of the studies. Participants were diverse, with the most common samples involving overweight or obese adults (*n* = 6),^24–29^ university students (*n* = 5)^30–34^ and shoppers (*n* = 2).^35,36^ Further details on participant demographics and study characteristics are provided in Supplementary Content 2 and 3.

All 38 studies were positive for the 2 screening questions of the MMAT. Three studies^37–39^ employed qualitative methods, and all were deemed to be of strong quality. Among the seven randomised controlled trials (RCTs), one^29^ was of strong quality, five^28,30,34,40,41^ received a mix of ‘yes’, ‘no’, and ‘unknown’ ratings, and one^42^ was not applicable throughout the screening tool. Six studies were non-randomised trials, four^43–46^ of which were of strong quality. One study^36^ received a ‘no’ for question 3.4, and another^7^ contained an ‘unknown’ response. Twelve studies were quantitative descriptive studies. Of these, three were found to have strong quality, four received either a ‘no’ or ‘unknown’ rating, and five contained ‘not applicable’ responses for one or more questions. Among the ten studies^25,32,35,47–52^ that employed mixed methods, eight had strong quality. One study^51^ contained two ‘no’ responses, and another^53^ had two ‘unknown’ responses and one ‘no’ response. Further details of the MMAT scores are provided in Supplementary Content 4.

### Study characteristics

Among the 38 studies, four^53–56^ specifically addressed podcast development, while the remaining 34^7,8,20,24–51,57–60^ focussed on podcast implementation and evaluation. Of the 34, *n* = 8 were pre-post studies^27,32,35,36,43–45,47^; *n* = 11 were randomised trials^24–26,28–30,40–42,48,60^; *n* = 2 were non-randomised trials^7,46^; *n* = 2 were qualitative^37,39^; *n* = 4 were cross-sectional^8,20,58,59^; *n* = 1 involved both podcast development and a RCT^51^; and *n* = 6 used various other designs (including audit, quasi-experimental, mixed methods, and participatory action research).^31,34,38,49,50,57^

The podcasts covered a range of subjects, with predominant themes including general health (*n* = 5),^53–56^ weight loss (*n* = 5),^31,44,54,58,59^ and mindfulness or meditation (*n* = 4).^24,25,27–29,60^ Refer to Supplementary Content 4 for a detailed breakdown of additional topics.

#### Overview of podcast development studies (*n* = 4)

Four studies^53–56^ detailed podcast development. An overview of podcast development studies is shown in Supplementary Content 3. They used various educational and behaviour change theoretical frameworks: Balls-Berry et al.^54^ utilised Kirkpatrick's Model, Level 1 and 2; Leite et al.^55^ drew upon Paulo Freire's work, while Mugisha et al.^56^ referenced the Health Belief Model, Technology Acceptance Model, and the Theory of Planned Behaviour and Conde-Ferraez et al.^53^ refrained from referencing theories.

The research and development outcomes were varied and tailored to each specific study. These included the availability of COVID-19 informational materials in the Mayan language, the exploration of previously unaddressed Mayan language topics, studies on comorbidities within the Yucatan Peninsula population, the process of creating podcast content and a review of the literature on the needs surrounding sexual and reproductive health education. Development outcomes were also tailored to the individual studies, and included the formulation of podcast themes, the use of social media platforms like SoundCloud, Twitter and Facebook for promotion, and the tracking of audience engagement metrics such as downloads, shares, and reactions. Furthermore, these studies involved monitoring how many times podcasts were played, analysing the geographical distribution of listeners, evaluating the podcasts against theoretical frameworks, and ensuring the material's quality in terms of its content, functionality, visual appeal and auditory environment.

#### Overview of podcast implementation and evaluation studies (*n* = 34)

An overview of studies that involved implementation and evaluation of podcasts is shown in Supplementary Content 4. Twelve studies^8,20,24–27,29,45,49,50,57,59^ utilised pre-existing podcasts, while seven^7,36,38,40,44,47,51^ created podcasts specifically for their research. Additionally, two studies^28,32^ integrated both newly created and pre-existing podcasts, and one study^46^ involved participants in co-creating study-specific podcasts. The origin of podcast creation remained unclear in 11 studies.^30,31,33–35,37,39,41,43,49,61^ Additionally, one study^58^ didn't focus on podcast creation per se but rather assessed the engagement with various technological tools for health information dissemination.

Various theoretical frameworks were incorporated into these studies. While 14 studies^8,20,30–32,40,41,43–45,47,51,57,59^ did not explicitly align with any theories, others incorporated established theories. Social cognitive theory was referenced in four studies,^24,26,29,42^ with self-determination theory^48^ and theory of reasoned action^35^ each used in two studies. Furthermore, eight studies^27,28,34,37,38,50,58,60^ incorporated multiple theoretical frameworks in their analysis.

### Aim 1: effectiveness for changing health and health-related outcomes

An overview of results from intervention studies that utilised podcasts as the main component for influencing health-related behaviours is detailed in Table 2. In the domain of diet and nutrition, the studies highlighted significant improvements in Omega-3-rich food perceptions and purchases (*n* = 3),^35,36,46^ folate knowledge (*n* = 1),^46^ and daily calorie intake (*n* = 1),^27^ with mixed results observed for fruit and vegetable consumption (*n* = 2).^27,28^ For physical activity, significant improvements were reported for vigorous activity (*n* = 1),^28^ self-monitoring and total activity (*n* = 1),^60^ whereas no significant changes were reported for moderate activity (*n* = 1).^27,28^ Regarding physical health outcomes, significant improvements were noted in weight and BMI (*n* = 2),^28,60^ whereas no changes were observed in two other studies (*n* = 2).^26,27^ In the mental and emotional health category, increased birth satisfaction (*n* = 1),^40^ was reported, while there was no change in depression and anxiety levels (*n* = 3).^30,40,44^ Lastly, disease management and health knowledge outcomes showed improvements in the ability to assess treatment options (*n* = 1),^51^ and in diabetes and weight-loss knowledge (*n* = 2),^28,45^ with no significant changes detected in overall cancer symptoms (*n* = 1).^44^

**Table 2.** Overview of results from intervention studies that used podcasts as the main intervention component on health-related behaviours.

Outcome	Results
**Diet and nutrition**	
Fruit and vegetable consumption	↑ *n* = 1 ↔ *n* = 1
Perceived ability to buy Omega-3-Rich Food	↑ *n* = 1
Perceived importance regarding buying Omega-3-Rich Food	↑ *n* = 1
Purchase of Omega-3-Rich Food	↑ *n* = 1
Folate knowledge	↑ *n* = 1
kcals/day	↑ *n* = 1
Folate consumption	↔ *n* = 1
Fatty foods consumption	↔ *n* = 1
Bites/day	↔ *n* = 1
**Physical activity**	
Vigorous physical activity	↑ *n* = 1 ↔ *n* = 1
Moderate physical activity	↔ *n* = 2
Walking	↔ *n* = 2
Sitting	↔ *n* = 2
Days per week of physical activity self-monitoring	↑ *n* = 1
Total physical activity	↑ *n* = 1
**Physical health**	
Bodyweight	↑ *n* = 2
Body mass index	↑ *n* = 2
Weight loss	↔ *n* = 2
Physical health	↔ *n* = 1
**Mental and emotional health**	
Depression	↔ *n* = 3
Anxiety	↔ *n* = 2
Birth satisfaction scores	↑ *n* = 1
Personal control during childbirth	↑ *n* = 1
Mental health	↔ *n* = 1
Self-harm	↔ *n* = 1
Pain intensity	↔ *n* = 1
Sleep disturbance	↔ *n* = 1
Mindfulness	↔ *n* = 1
Life satisfaction	↔ *n* = 1
**Disease management and health knowledge**	
Ability to assess claims about treatment effects	↑ *n* = 1
Diabetes knowledge	↑ *n* = 1
Weight-loss–related knowledge	↑ *n* = 1
Overall cancer symptoms	↔ *n* = 1

^↑^ Statistically significant improvement; ^↔^ No significant change. References on following page.

### Aim 2: usage patterns and engagement

#### Listening statistics

Engagement and experience with podcasts were examined in seven and four studies, respectively, with seven studies covering both aspects. Cai et al.^40^ reported 82.1% participation in the podcast education group, while Huberty et al.^44^ noted 83.3% weekly completion of prescribed podcasts, averaging 103.2 ± 29.5 minutes. Biber et al.^43^ found an average listening time of 124 ± 17.86 minutes, and Carrotte et al.^20^ observed 62.3% of participants listening to at least four podcast episodes. Turner-McGrievy et al.^33^ reported significantly more time spent on podcasts than web content (*p* < 0.001), and Laird et al.^41^ noted 62% completion of prescribed meditation in the podcast group compared to 71% in the Calm (app) group.

#### How people listened

Turner-McGrievy et al.^28^ found that during podcast listening, most participants were sedentary, with 44.8% at their desk and 22.4% at home. The majority (53.7%) preferred listening at home, followed by in the office or at work (20.9%) and while exercising or walking (13.4%). Karing et al.^30^ found that there was no significant difference in the commitment to home practice between participants who used mindfulness podcasts and those who engaged with mindfulness through videoconferencing.

#### Topics

Regarding mental health-themed podcasts, those focusing on interviews with people with lived mental health issues were reported as the most popular type. Caoilte et al.^8^ identified that individuals with a history of accessing mental health services were more likely to listen frequently to mental health podcasts, with previous struggles with mental health significantly correlated with listening frequency. Additionally, participants with higher education levels tended to listen more frequently to mental health-related podcasts for entertainment compared to those with lower levels of education.

#### Podcast download statistics

Turner-McGrievy at al.^27^ reported consistent mean weekly podcast downloads throughout their intervention, with week 1 having 1.6 ± 0.2 downloads, week 2 having 1.1 ± 0.3 downloads, week 3 having 1.2 ± 0.3 downloads, and week 4 having 1.1 ± 0.3 downloads. In another study, Turner-McGrievy et al.^29^ observed more podcasts downloaded by the app group (31.0 ± 2.7) compared to the comparison group (26.1 ± 2.8, *p* = 0.22), although not statistically significant. However, Dunn et al.^24^ found there was no significant correlation between the number of podcasts downloaded and weight change.

#### Technology and podcasts (e.g. apps and podcast adoption):

Militello et al.^50^ reported that the app used in their intervention deployed 239 podcasts across 17 participants within a 2-week time frame. LeRouge et al.^58^ investigated baby boomers’ use of technology for health and found that they were less likely to use podcasts compared to older adults. Among the five technologies studied, baby boomers responded most favourably towards smartphones and podcasts.

### Aim 3: perspectives towards health podcasts

#### Subjective user experiences and information delivery

Across diverse studies, subjective user experiences regarding podcasts were examined, emphasising factors like information clarity, authenticity and validity. Shaw et al.^39^ noted that the inclusion of personal stories enhanced authenticity, with the speaker's tone and emotions adding validity to participants’ experiences. Similarly, Militello et al.^50^ found a positive impact of anecdotal experiences when supported by medical data. Two other studies reported on preferences for podcasts with objective data, clear information delivery, and succinct content.^35,48^

#### Mindfulness and meditation podcasts

Limited data on mindfulness/meditation podcasts revealed participant preferences and experiences. A study reported varying preferences between face-to-face and digital sessions, citing factors like distraction, privacy benefits and calming effects. Suggestions for improvement included enhancing sound clarity, adjusting volume, adding background music and using a female narrator.^35^

#### Improvements and additional comments

User feedback encompassed suggestions for podcast enhancements, such as sound quality, volume adjustments and narrative elements. Practical application challenges were noted, with some participants reporting finding podcasts tedious or confusing.^35^ Preferences for podcasts varied within multi-component interventions, with some participants viewing podcasts as the best part, while others considered them less favourable.^48^ Participants expressed interest in specific topics, alternative solutions and information validating their prior knowledge and experiences.^50^

#### Listener motivations and mental health focus

Carotte et al.^20^ highlighted prevalent motivations for engaging with mental health podcasts, emphasising the desire to understand mental health issues, acquire supportive strategies and explore personal interests. Carotte et al.^20^ identified distinct patterns among participants with mental health struggles, demonstrating a lower focus on acquiring new information but a higher inclination towards using podcasts for emotional acknowledgment, particularly among those who accessed mental health services.

#### Satisfaction, trustworthiness and technological considerations

Three studies^30,41,44^ reported on satisfaction, with Karing et al.^30^ and Huberty et al.^44^ both reporting high levels of satisfaction with the implemented health-related podcast. Conversely, Laird et al.^41^ reported that participants were more satisfied using the Calm app, than those who used the podcast alone.

Both Weib et al.^34^ and Semakula et al.^51^ reported that participants had positive views on the podcasts delivering health information. While Huberty et al.^44^ reported that participants found that there were technical and functionality difficulties with the health education podcast app, with 33.3% of the participants having issues accessing specific podcasts.

Three studies^31,34,51^ reported on the trustworthiness of health podcasts. Semakula et al.^51^ reported that participants trusted the information that they learnt from the podcasts. While Kirkpatrick et al.^31^ and Weib et al.^34^ reported that expert sources were the most highly trusted for the delivery of health information.

#### Realism, representation and context

Shaw et al.^39^ highlighted the significance of real women sharing stories, providing helpful and informative content. Participants valued narratives from laypeople over health professionals or doctors, appreciating the medical context provided by the host. While a pharmaceutical sponsor was off-putting, participants found the podcast more representative and multi-dimensional than written information. Though some felt unrepresented due to differences in menopause perceptions, symptoms discussed, and storyteller demographics, participants recognised the podcast's informational value and its contribution to a collective sense of womanhood.

## Discussion

### Overview of key findings

This scoping review set out to describe the current state of scientific evidence on the impacts, engagement and perceptions of health-related podcasts. We identified 50 articles reporting on 38 unique studies involving a total of 5094 participants. These studies, published from 2004 onwards, spanned a broad spectrum of subjects, most commonly general health, weight loss, and mindfulness or meditation. The review uncovered diverse applications and evaluation of podcasts, which included both pre-existing podcasts and content specifically created for research purposes. In terms of the effectiveness of podcasts in promoting health and health-related behaviours, some studies reported positive changes in diet and nutrition, physical activity, and specific health outcomes like weight and BMI, and others found no significant improvements. Engagement was reported in several different ways, but generally suggested high participation rates that compared favourably to alternative delivery platforms. Listeners predominantly engaged with podcasts at home or work, with the most common motivations centred around acquiring health information, understanding mental health issues and seeking entertainment.

This review found preliminary evidence that podcasts may be effective in enhancing health knowledge and facilitating changes in health behaviours, such as increased physical activity and improved dietary habits, leading to outcomes like weight loss. However, there was less evidence of impact of podcasts on psychological outcomes, such as depression and anxiety, with all three studies focusing on these outcomes reported no significant changes. The variation in effectiveness may be because podcasts are well-suited for delivering informational content. This may be sufficient to support behaviour change related to physical activity and diet, but not for mental health, which may require a more personalised or more introspective approach. It's important to acknowledge, however, that the evidence base for the impact of podcasts is relatively small and consists mainly of single-group studies and a small number of mostly small-scale RCTs. Consequently, the current evidence does not allow for definitive conclusions about the effectiveness of podcasts for specific health outcomes.

Our review results indicated that engagement with health-related podcasts is generally high, suggesting that podcasts are an engaging medium for health information and behaviour change interventions. For example, engagement metrics suggested that participants generally listening to 1–2 episodes per week and the majority listening to over half of the assigned podcasts. This finding aligns with broader trends in podcast consumption, though falls short of the average listening time of 9.0 hours per week reported among general podcast listeners in 2023.^1^ It is possible that the intent and context of listening to health-related content versus general content may differ; for health content – listeners might engage with podcasts with a specific purpose or need in mind, potentially leading to more focused but less time-consuming engagement compared to general leisure listening. The high engagement with health podcasts would appear to be an advantage in comparison to other health intervention modalities. It is somewhat surprising how little attention podcasts have received in the scientific literature, compared with other digital approaches, such as smartphone apps, which have been around for a similar length of time, yet received vastly more research attention.

Findings related to users’ perspectives on health-related podcasts revealed insights about the medium's appeal as well as potential areas for enhancement. Listeners generally expressed positive views on podcasts, appreciating the convenience and accessibility they offer. They valued the ability to access health information at their own pace and in a private setting, which aligns with the increasing consumer demand for health resources that are not only informative but also adaptable to individual lifestyles.^62^ The authenticity and relatability of health podcast content emerged as important factors. Personal stories and experiences shared within podcasts were highlighted as particularly impactful, suggesting that narrative-driven content enhances the relatability of health information. However, despite the overall positive reception, listeners also pointed out areas for improvement. These include the desire for higher sound quality, more varied content delivery (e.g. incorporating music or sound effects), and the inclusion of expert insights to bolster the informational value of podcasts. Such feedback suggests that a balance between maintaining the informal, relatable nature of podcasts and enhancing their educational credibility is needed.

## Strengths and Limitations

Our study is the first study to review the evidence regarding health podcasts for the general population. Strengths of our study include its highly comprehensive approach, encompassing health podcasts’ impacts, engagement and user perspectives. We adhered to a highly robust scoping methodology, which followed PRISMA-ScR guidelines^17^ and the Arksey and O'Malley framework.^18^ Furthermore, the study went beyond standard practice for scoping reviews, by appraising the included studies’ risk of bias.

Study limitations must also be acknowledged. We focus on English language publications; it is possible that relevant studies have been published in other languages have been missed (though we didn’t identify any during our database searches). Further study limitations relate to the current state of the scientific body of evidence regarding health podcasts. At present, the efficacy data predominantly consists of single-group studies and small RCTs, which introduces potential biases and limits the generalisability of the findings. Additionally, the high level of heterogeneity in terms of study designs, podcast topics and formats, and outcome across the included studies limits the ability to draw firm conclusions. Finally, it should be noted that a further limitation is the unknown quality of health advice in podcasts, as anyone can claim to be an expert and start a podcast, posing a potential risk of harm if the information provided is inaccurate or misleading. Another potential limitation of our review is its restriction to published data only. However, by including only published data, we ensure that our review is based on high-quality, peer-reviewed studies, which enhances the reliability of our findings.

## Future Research Directions

Findings from this review show that health podcasts, achieve strong engagement, are well received, and can lead to measurable changes in health behaviour and health outcomes. Given current evidence and findings from this review, the following topics appear to be valuable areas for future research and practice:

1. Given that podcasts achieve relatively high engagement, it appears they are currently being underutilised as a health promotion tool. Increased effort to harness podcasts for health promoting activities appears warranted.
2. Given the huge and ever-increasing volume of health podcast content, it will be challenging for listeners to identify the podcasts that are most relevant and beneficial to them. It is possible that artificial intelligence may be able to curate content based on individual's preferences and health goals.
3. Studies that experimentally investigate the impact of different podcast formats, such as narrative storytelling versus informational lectures, are needed to determine the impact on listener engagement, and learning and behavioural outcomes.
4. Large-scale RCTs with long-term follow-up are needed to provide more rigorous evidence regarding podcasts’ short- and long-term health impacts.
5. Research is needed to determine the optimal design of podcasts aimed at improving mental and emotional well-being. This could include studies on podcast that incorporate therapeutic techniques, or that offer podcasts as part of a multi-component intervention approach.

## Conclusion

In conclusion, this scoping review highlights the promising potential of health-related podcasts for health promotion. While the current evidence base is limited, it collectively indicates that health podcasts are highly engaging, well received and can positively impact health behaviours and outcomes. There is a clear need for future research to fully leverage the unique benefits of podcasts for public health. Large-scale, methodologically robust research is needed to substantiate the long-term impacts of health podcasts, and maximise their potential as a tool in health education and behaviour change interventions.

## Supplemental Material

sj-docx-1-dhj-10.1177_20552076241288630 - Supplemental material for Podcasts as a tool for promoting health-related behaviours: A scoping review Supplemental material, sj-docx-1-dhj-10.1177_20552076241288630 for Podcasts as a tool for promoting health-related behaviours: A scoping review by Bethany Robins, Tessa Delaney, Carol Maher and Ben Singh in DIGITAL HEALTH

sj-docx-2-dhj-10.1177_20552076241288630 - Supplemental material for Podcasts as a tool for promoting health-related behaviours: A scoping review Supplemental material, sj-docx-2-dhj-10.1177_20552076241288630 for Podcasts as a tool for promoting health-related behaviours: A scoping review by Bethany Robins, Tessa Delaney, Carol Maher and Ben Singh in DIGITAL HEALTH
