# Supplementary material for: Podcasts as a tool for promoting health-related behaviours: A scoping review
Source: Digit Health. 2024 Oct 13;10:20552076241288630. doi: 10.1177/20552076241288630 (PMC11472369; doi:10.1177/20552076241288630)
Supplement: sj-docx-1-dhj-10.1177_20552076241288630 - Supplemental material for Podcasts as a tool for promoting health-related behaviours: A scoping review [file sj-docx-1-dhj-10.1177_20552076241288630.docx]

Supplementary content 1. Search terms.

| Search Category | Search Terms |
| --- | --- |
| Podcast | “podcast*” OR “pod cast” OR “audiocast” OR “audio cast” OR “digital narrativ*” OR “digital story” OR “digital stories” |
| Health-related behaviour and outcome | “health*” OR “human health” OR “weight” OR “lifestyle” OR “behaviour” OR “health behaviour” OR “health literacy” OR “functional health” OR “diabetes” OR “obesity” OR “heart disease” OR “cancer” OR “body weight” OR “inflammation” OR “oxidative stress” OR “dyslipidemia” OR “chronic condition*” OR “dementia” OR “mortality” OR “death” OR “treatment” OR “smoking” |
| Physical activity and sedentary behaviour | “physical activity” OR “physically active” OR “physical inactivity” OR “physically inactive” OR “exercise” OR “fitness” OR “exercis*” OR “sport*” OR “walk” OR “walking” OR “sedentary” OR “sitting” OR “television time” OR “TV time” OR “screen*” OR “screen time" OR "sedentary time" OR "sedentary lifestyle" OR “screen-time” OR “active transport*” OR “active transit” OR “active travel” OR “commut*” OR “active commuting” OR “bicycle” OR “bicycling” OR “bike” OR “biking” OR “active living” OR “active-living” |
| Sleep | “sleep” OR “insomnia” |
| Nutrition | “diet*” OR “nutrition*” OR “eat*” OR “eating behaviour*” OR “food” OR “food intake” OR “calorie” |
| Wellbeing | “mental health” OR “depression” OR “mediat*” OR “health” OR “wellbeing” OR “well being” OR “well-being” OR “wellness” OR “ill$” OR “anxiety” OR “anxious” OR “depress$” OR “quality of life” OR “stress*” OR “psychological stress” OR “depress*” |

Supplementary content 2. Overview of podcast development studies (n=4).

| **Study** | **Study design** | **Participant characteristics** | **Study aims** | **Target health-related behaviour of the podcast** | **Podcast overview** | **Outcomes** |
| --- | --- | --- | --- | --- | --- | --- |
| Balls-Berry 2018, USA | Descriptive (podcast development) | NA | Establish and publicise a podcast library on social media for sharing health research with the public. | Health-related | Series of 45 episodes with an average duration of 22 minutes and 19 seconds (±12 minutes and 25 seconds). Top 5 topics: injury prevention, immunizations, overweight/obesity, mental health, and financial stress. | - Develop podcast topics, promote on social media (SoundCloud, Twitter, Facebook), track audience metrics (downloads, shares, reactions), monitor play count, episode length, and listener locations for comprehensive analysis. |
| Conde-Ferráez 2022, Mexico | Descriptive (podcast development) | N/A | Generate and make accessible health communication materials on the COVID-19 pandemic in Mayan language. | Covid-19 | 3x 30-second "Stay at home" messages in Mayan language were produced. The study created 11 video clips (20 seconds to 2 minutes 44 seconds) in .mp4 format for social networks. Additionally, six podcast episodes, around 20 minutes each, were produced in both Mp3 and wav formats, covering health, mental health, reactivation, mourning, community support, and COVID-19 myths. | - Exhaustive search: Number and type of COVID-19 materials available in the Mayan language and any topics not covered in the Mayan language. - Documentary research: Comorbidities in the Yucatan Peninsula. - Conceptualisation of podcast content |
| Leite 2022, Brazil | Podcast development and validation | n=60 teens, Female: 61.7%, 17±2.5 y | Construe and validate a podcast for teen sexual and reproductive health. | Teen sexual and reproductive health education | Four episodes were created covering topics such as sex and sexuality, contraceptive methods, HIV/AIDS, and other STIs. Each episode has a duration ranging between 8 to 11 minutes. | - Literature review – sexual and reproductive health education needs. - Validation of the material – content, functionality, appearance and sound environment. |
| Mugisha 2022, Rwanda | App/podcast development | N/A | Describe development of a mobile app for health education for youth in Rwanda. | Adapted “Health Choices” book | The developed app consists of 10 podcasts designed to educate youth about treatment claims and guide them in weighing the advantages and disadvantages of treatments before making choices. | - Podcasts created, based on 3 conceptual theories. |

Supplementary content 3. Overview of studies evaluating podcasts and interventions involving podcasts (n=34).

| **Study** | **Study design** | **Participant characteristics** | **Study aims** | **Target health-related behaviour of the podcast** | **Podcast overview** | **Outcomes** |
| --- | --- | --- | --- | --- | --- | --- |
| Bangia 2014, USA | Repeated measures design | n=56 shoppers  Female: 79%  41±15 y | Assess the effects of the podcast’s use at point of purchase. | Omega-3 information | Single 5-minute podcast, accessed via MP3, guides to omega-3 foods, store-aligned, emphasizes DHA/EPA-rich foods, particularly seafood. | - Pre- and post-intervention interviews assessed Theory of Reasoned Action constructs, pre-focusing on prior podcast use, and post-exploring likes, dislikes, and improvement suggestions. Visual verification of purchased foods on the interview day was also conducted. |
| Bangia 2017, USA | Repeated measures, 12m | n=173 shoppers  Female: 76%  50.4±13.8 y | Assess the impacts associated with a grocery tour point-of-purchase intervention using podcasts about omega-3 fatty acid rich food. | Omega-3 information | 10 podcasts (main intervention) ranging from 38 seconds to 6 minutes and 20 seconds. Cover topics include omega-3 rich foods, their types, health benefits, and food sources. | - The number of omega-3–rich food purchases made according to food or food category. |
| Biber 2021, USA | Pre-post, 8w | n=20 university employees  Female: 80%  42.7 ±13.2 y | Evaluate the effectiveness of self-compassion intervention within a workplace PA program. | Self-compassion  Meditation | A 7-week self-compassion podcast intervention, running alongside Desire2Move procedures, began at week 2. The intervention consists of 7 podcasts, one per week, with durations ranging from 5 to 24 minutes (average 17:57). The content covers various self-compassion topics and includes meditations, delivered as MP3 audio files via email. | - Self-compassion (self-compassion scale-short form) - Duration and mode of PA (MapMyRun) - Intervention adherence – frequency and length of podcast use (tracked via private YouTube account) |
| Cai 2023, USA | RCT | n=153 pregnant women    Podcast education group: 30.4 ±5.3 y    Usual education:  29.3 ±6.1 y | Assess the effect of a lab or education podcast on personal perception of control and patient childbirth satisfaction. | Labour education | 7 podcast episodes, each addressing specific labour-related topics such as anaesthesia, complications, normal labour, caesarean delivery, second stage, postpartum recovery, and induction. Episodes are 10-20 minutes long, designed for accessibility on podcatchers, ensuring content is at or below a 6th-grade reading level. | - Personal control during childbirth (10-question Labour Agentry Scale) - Birth satisfaction (birth satisfaction survey) - Postnatal depression (Edinburgh Postnatal Depression Scale) - Podcast use metrics |
| Caoilte 2023, Ireland | Cross sectional | n=722  Female: 69% | Explore the motivations and experiences of mental health-related podcast listeners. | Mental health | The main intervention involves podcasts addressing topics related to mental health and psychology. | - Engagement with mental health podcasts, usefulness of resources and ratings of statements linking to podcasts. - Listening frequency and reasons for listening |
| Carrotte 2023, Australia | Cross sectional | n=629  Female: 71.1%  28.6 ±11.4 y | Examine demographics, behaviours, and motivations of mental health podcast listeners, evaluating whether they differ in attitudes and knowledge from general audiences. | Mental health | One podcast in the last 12 months (on any topic). | - Podcast listening - Stigma, knowledge and response style – stigmatizing attitudes and discriminatory intentions (Attribution Questionnaire - Short Form), internalised stigma (Internalised Stigma of Mental Illness – short form), mental health knowledge (Mental Health Knowledge Schedule), socially desirable response style (Balanced Inventory of Desirable Responding – Short Form). |
| Dreer 2021, Germany | Non-RT, 16w | n=117 student teachers  Female: 82%  25.5 ±3.4 y | Evaluate the impact of an audio podcast intervention on the overall and job-related well-being of student teachers. | “Positivity for student teachers” | The intervention comprises nine episodes, each lasting less than 20 minutes. The content is divided into knowledge acquisition (episodes 1-4), practicing (episodes 5-7), and reflection (episodes 8-9). The complete intervention is delivered through Moodle within an existing course structure, with the podcast serving as the core element. | - General well-being, life satisfaction and happiness (German version of the Satisfaction with Life Scale and the Subjective Happiness Scale). - Job related well-being (scales measuring job satisfaction, teacher engagement, emotional exhaustion) |
| Dunn 2019, USA | RCT, 6m | n=43 overweight or obese adults  Female: 90.5%  42.4 ±12.4 y | Compare the efficacy of a mobile photo dietary app to a calorie-tracking app on tracking frequency and weight loss in a remote behavioural weight-loss intervention. | Behavioural weight-loss | A six-month intervention involved twice-weekly podcasts incorporating behavioural weight-loss techniques. A total of 48 podcasts were distributed via email to both control and intervention groups. The podcasts were used in conjunction with different apps, depending on whether participants were in the intervention or control group. | - App interface tracked self-monitoring frequency. - Date, time and content included objectively measured self-monitoring frequency. - Number of podcasts downloaded. - Weight |
| Fine 2022, USA | Audit | N/A | Review the availability, content, and authorship of podcasts on the topic of fertility and to compare those hosted by physicians and patients. | Fertility | 133 podcasts, 84 active (2009–2020), mostly from USA (92%), Australia (4%), the UK (3%), and Ireland (1%). Primarily for patients (99%), covering patient experience (62%), fertility awareness (30%), third-party reproduction (13%), and physician education (1%). Hosts include patients (40%), holistic health professionals (28%), physicians (16%), and third-party agencies (11%). | - Podcast title, subject category, host type, target audience, country association, published date, number of episodes, episode frequency, number of ratings and status. |
| Goodridge 2021, Canada | Pre-post, 12w | n=29 caregivers  Female: 90%  59.6±11.3 y | Explore a 12-week mobile support program's feasibility and evaluate its efficacy on caregiver burden, coping styles, and well-being. | Mindfulness-based self-compassion | The intervention comprises 14 podcasts cantered on mindfulness-based self-compassion, delivered via a mobile app. Example topics include "Who should practice mindfulness and self-compassion?" "Introduction to loving-kindness practice," and "The power of gratitude." | - Caregiver burden (Burden Scale for Family Caregiving) - Coping styles (Brief-COPE) - Emotional well-being (World Health Organisation-5 Well-Being Scale) - App acceptability, practicality and implementation |
| Hales 2016b, USA  Hales 2017  Hales 2016a | RCT, 3 m | n=51 overweight or obese adults  Female: 82%  46.2±12.4 y | Assess an app's efficacy for weight management through social support and self-monitoring, comparing it to a commercial diet and PA tracking app for overweight and obese adults. | Nutrition and exercise information presented in podcasts  Podcast audio diary  Podcast weight-loss goal | The intervention includes two theory-based podcasts per week, focusing on nutrition, PA, and weight loss. The podcasts are accompanied by the use of the Social POD app, which features components related to diet, PA, and a journal. Participants receive these resources via email. | - Weight loss - Group differences in BMI, caloric intake and expenditure, social support for diet and exercise behaviours, self-efficacy for weight loss and outcome expectations of treatment |
| Huberty 2020, USA | Pre-post, 12w | n=39 myeloproliferative neoplasm cancer patients  Female: 94.9%  56.1±10.9 y | Beta-test a health education podcast via smartphone app for potential use as a robust comparator in future app-based meditation intervention studies. | Health education | 60 minutes per week (main component). Average 22 minutes x 2-3 podcasts per week | - Podcast use - Satisfaction - Changes in health and cancer-related symptoms |
| Johnson 2012, USA | Pre-post (1 session) | n=30 African American men  Female: 0%  Mean: 28 y | Explore the effectiveness of an emergent technology (podcasting) for use in educating inner-city, African American men about diabetes | Diabetes | Diabetes education intervention specifically designed for African American men. It delivers knowledge through a laptop via headphones, with each session lasting 14 minutes. | - Diabetes knowledge |
| Karing 2022, Germany | RCT, 7w | n=50 university students  Videoconferencing group:  Female: 80%  23.2 ±2.9 y  Podcast group:  Female: 88%  25.0 ±8.3 y | Compare guided mindfulness intervention via videoconference with unguided PODs training, evaluating program satisfaction, attendance, compliance, and their impact on intervention outcomes. | Mindfulness | The intervention involves a weekly podcast with elements such as a theoretical introduction, mindfulness exercises, and explanations, along with reflexive questions. Delivery is facilitated through the SoSci Survey platform. | - Trait mindfulness - Depression - Anxiety - Life satisfaction (subjective well-being) - Feasibility - program satisfaction, recommendation of the online training, online course attendance and compliance with homework assignments. |
| Kirkpatrick 2021, USA | 3x2 within-subjects factorial | n=113 university students  Female: 82%  19.9±1.6 y | Explore the impact of source type and message relevance on message processing and persuasion in the context of health podcasts, as a health promotion tool. | Health podcasts | 18 one-minute excerpts, with participants listening to six each. Topics cover alcohol poisoning, stress/anxiety, meningitis, early-onset Alzheimer’s disease, heart disease, and osteoporosis. The intervention is delivered through a computer with headphones. | - Perceived source competence, source trustworthiness, message effectiveness, health behaviour intention, podcast download intention. |
| Knudsen 2022, Denmark | Qualitative | Focus groups: n=16 patients and health professionals  Feasibility test: n=10  Females: 70%  Mean: 58 y | Develop an e-learning education program targeting patients w/ rheumatoid arthritis. | Patient education | The program incorporated entertainment-education elements delivered through podcasts. Seeking participant opinions, the podcasts were subsequently integrated into the patient education program. | - Focus group themes: knowledge of rheumatoid arthritis, the disease course and the prognosis, medical treatment, a new life situation. - Feasibility testing: general impression of the program, user perception, comprehensibility of the contents, reader-friendliness, layout/design, length and number of presentations. |
| LaBrosse 2013, USA | Non-RT, 15w | n=44 school students  Intervention:  School A: 42% female  School B: 85% female    Control:  School 1: 57% female  School 2: 25% female | Develop a health belief model educational intervention to enhance folate-rich food knowledge and consumption in adolescents, fostering positive beliefs. | Folate information | Participants created 30-90 second podcasts incorporating class concepts. Completed podcasts were played in class weekly for 8 weeks, forming the main intervention, which included the process of podcast development. | - Participants folate intake, knowledge and beliefs. |
| Laird 2022, USA | RCT, 4w | n=58 middle-aged adults  Calm group (n=32)  Female: 62.5%  52.1±6.7 y old  Podcast group (n=26),  Female: 69.2%  50.8±6.9 y old | Assess the feasibility and effects of the Calm meditation app on stress reduction, mental health, behaviours, and COVID-19 perceptions in stressed middle-aged adults. | Health and well-being | Participants engage in a 10-minute daily podcast, alternating between meditation and educational content, delivered through either a control or intervention app. | - Feasibility of a consumer-based meditation app. - Self-Reported: perceived stress, anxiety, depression, mindfulness, PA, eating habits, general coping and COVID-19 perceptions. |
| Lauricella 2014, Canada | Pre-post, 1w | n=40 university students  Female: 68%  Mean: 20 y | Assess the preferred method of delivery (face-to-face in a group, or digitally via mp3) for undergraduates learning mindfulness meditation. | Guided meditation | Traditional body scan technique, via personal devices | - Method of delivery they preferred and why, how participants felt immediately after the sessions. |
| LeRouge 2014, USA | Cross sectional | n=469  Young group (n=72)  Female: 73.6%, 35.3±7.9 y  Baby boomer group (n=258), Female: 55.4%  56.9±4.8 y  Older group (n=139)  Female: 43.9%, 73.2±6.2 y | Examine baby boomers' technology readiness and barriers. Identify preferred technologies and explore obstacles adoption of health tech. | Health information | N/A | - Boomer readiness, readiness comparisons among age groups, boomer barriers, barrier comparisons among age groups |
| Mailey 2016, USA | RT, 16w | n=69 working mothers  35.9±5.1 y | Explore the feasibility and effectiveness of delivering an entirely web-based version of the Fit Minded intervention to working mothers. | Well-being | A weekly 20–30-minute podcast is utilized in both the standard and enhanced conditions, addressing themes of self-renewal, stress reduction, work-life balance, and mindfulness. | - PA, self-worth, exercise motivation, self-efficacy, program evaluation. |
| Mailey 2019, USA | Quasi-experimental, 10w | n=231 military spouses  IBNA group (n=119)  Female: 100%  31.9 ±6.1 y  Control group (n=112)  Female: 100%  33.1 ±6.3 y | Investigate the impact of an interactive, theory-based web intervention compared to a generic educational one on mental health outcomes and health behaviours in military spouses. | Health and well-being | The key element of the IBNA intervention involves 3-5 weekly podcasts, with a focus on PA and diet, along with 1-3 podcasts related to personal growth or emotional well-being. The podcasts are accessible via a website. | - Mental health outcomes - PA - Diet - Engagement - Program evaluation |
| McCall 2022, USA | Cross-sectional | n=313 parents  Female: 59.4%  34.2±7.1 y | Describe health-related technology use among parents of young children. | Health-related information, advice or support | N/A | - Sociodemographic characteristics, technology device ownership, access and use, search content, frequency and usefulness of parent health-related technology use. |
| Militello 2021, USA | Mixed methods, 2w | n=19 perinatal women  18-34 y: 84% | Assess the feasibility of delivering perinatal health education via voice among a group of perinatal women. | Perinatal health information | Retrieved episode content from Dr Mike PediaCast. 3-8 minutes long, accessed, via the app Main component of the intervention app | - App usage, podcast qualitative feedback (establishing/transitioning routines, expected norms and tempered expectations, provide key takeaways), customise and user preferences, privacy, family and friends, context and convenience. |
| Perry 2022, New Zealand | Participatory action research | Patient advisory group (n=7), Female: 86%; 20-60 y  Māori group (n=10)  Female: 80%, 30-70 y  Others (n=7; physiotherapists, researchers, health experts, Māori literacy expert) | Develop a co-designed, culturally responsive, online group-based pain management program (iSelfhelp) for people with persistent pain. | Relaxation audio podcast | The intervention includes relaxation audio podcasts featuring brief summaries of interventions (e.g., mindfulness) and fundamental concepts (e.g., fatigue). These podcasts are in downloadable MP3 format and are designed to complement modules within the app. | - Patient advisory group focus groups = video content, interactive texts, animations, metaphors, relaxation PODs, people’s stories, peer-support, online module structure. - Māori community-based focus groups - content should focus on not on ID but also family, use and English language on the website. |
| Semakula 2017, Uganda and Norway  Semakula 2019  Semakula 2020 | Podcast development + RCT, 7-10w | Podcast development: n=25 researchers, journalists, parents, other members of the public  Female: 56%  RCT: n=675 parents of primary school children  Podcast group (n= 334)  Female: 77%  Service announcement group (n=341)  Female: 76% | Podcast development: Design media resources to enable the public to appraise the trustworthiness about treatments and make informed choices.  RCT: Evaluate the effects of the podcast on parents of primary school children in Uganda to assess claims about the effects of treatments. | Informed health choices | Educational podcast development includes 8 episodes (5-7 minutes each), an intro, a 1-minute recap for every two main episodes, and a conclusion. Totalling 13 audio files in English and Luganda, it helps parents assess treatment claims. The RCT intervention spans 7-10 weeks, featuring 13 online episodes (5-10 minutes each) teaching treatment effect concepts, complemented by 12 additional learning resources. | - Podcast development involves creating key concepts for assessing treatment claims, generating teaching resources, and refining prototypes through qualitative interviews. - The RCT evaluates mean test scores, the proportion with passing scores, mastery of key concepts, and intended behaviors/self-efficacy. |
| Shaw 2022, United Kingdom  Edwards 2021 | Qualitative | n=30 women  48.8±7.42 y | Explore the impact of a podcast on women’s menopause knowledge, understanding and communication. | Menopause | "Menopause: Unmuted," consists of five episodes presenting real women's personal experiences with menopause in a storytelling format. Additionally, there is a sixth episode summarizing the content of the previous five episodes. | - Themes identified: openness and authenticity, strength of stories, power of the voice, relatability and representation, a community of women, feeling unrepresented. |
| Turner-McGrievy 2009, USA  Ko 2014 | RCT, 12w | n= Overweight and obese adults    Control (n=37)  Female: 81%  39.6±12.2 y  Enhanced (n=41)  Female: 68%  37.7±11.8 y | Examine if a weight-loss podcast designed with health behaviours theories, will produce greater weight loss than a currently available podcast that is not theory based. | Weight-loss | In the main intervention, both control and enhanced groups receive 2 podcasts per week for 12 weeks. The control group podcasts, averaging 18 minutes and 34 seconds, focus on weight loss discussions, emphasizing cognitive restructuring to avoid overeating for achieving a healthy weight. The enhanced podcast group, averaging 15 minutes and 42 seconds, is designed using constructs from social cognitive theory. | - Weight, food intake, PA and social cognitive theory constructs, perceptions of the intervention. |
| Turner-McGrievy 2013a, USA  *(#2887)* | RT | n=40 university students  Female: 90%  21.8±5.1 y | Test the effects of different media and examine both the direct effects of media on physiological arousal and to examine whether those effects are mediated by novelty and user control. | Weight-loss | The main intervention consists of a podcast with four components: 1. welcome, 2. first-person account, 3. nutrition and exercise information, and 4. a goal-setting activity. The podcast is approximately 12 minutes long, while the website contains the same information presented across four pages. | - Length of time participants spent on condition, change in skin conductance level arousal from baseline, use/information control questionnaire, novelty questionnaire and knowledge test score. |
| Turner-McGrievy 2017a, USA  *(#2900)* | Pre-post, 4 w | n=12 overweight adults  Female: 92%  54.1±11.3 y | Evaluate the user experience of the Bite Counter in a one-onth behavioural weight loss intervention, incorporating weekly face-to-face sessions, twice-weekly audio podcasts, and weekly behavioural challenges. | Used in weight-loss previously | In conjunction with the main intervention component includes the use of two 15-minute podcasts each week, previously employed to aid in weight loss in other studies. | - Dietary: Bites per day, Kcal per day, fruit and vegetable servings. - PA: Minutes of vigorous activity per week, minutes of moderate activity per week, minutes of walking per week, minutes spent sitting per weekday over the past 7 days. - Body Measurements: Body weight, weight loss from baseline. - Podcast Engagement: Mean number of podcasts downloaded by participants. |
| Turner-McGrievy 2017b, USA  *(#2914)*  Turner-McGrievy 2020 *(#2906)*  Turner-McGrievy 2019 *(#2908)* | RT, 6 m | n=81 overweight adults    App group (n=42)  Female: 83.3%  48.6±11.7 y  Bite group (n=39)  Female: 82.1%  47.5±12.3 y | Examine use of 2 different mobile dietary self-monitoring methods for weight-loss. | Weight-loss | The main intervention involves delivering behavioural weight-loss content through two podcasts per week, based on social cognitive theory and the Diabetes Prevention Program. Over the 6-month study, a total of 48 podcasts are administered to both groups. | - Weight change - Change in energy intake. - Change in total metabolic equivalents. - Total number of podcasts downloaded - Total number of days diet was recorded |
| Turner-McGrievy 2011, USA  Turner-McGrievy 2013b *(#2898)*  Turner-McGrievy 2014 | RT, 6m | n=96 overweight and obese adults  Podcast group (n=49)  43.2±11.7 y  Podcast+Mobile group (n= 47)  42.6±10.7 y | Explore the effectiveness of podcasting, mobile support communication, and mobile diet monitoring in aiding weight loss. | Described elsewhere | Both Podcast-only and Podcast + Mobile groups receive two podcasts weekly for the initial three months, and two Mini podcasts weekly for months 3–6. Participants access a group-specific podcast site for mobile or computer listening, covering behavioural weight loss topics emphasizing self-monitoring diet and integrating PA into daily routines. | - Body weight - Diet - PA - Self-efficacy |
| Weib 2023, Germany | 3x3 between-subjects design | n=320 university students  31.2±11.1 y | Explore whether media differ in their effectiveness to stimulate intention formation | The impact of nutrition on climate  Sugar in foods  The influence of nudges on eating behaviour | A 9-minute podcast intervention covers nutrition, climate, sugar, and dietary behaviour using social cognitive theory constructs. Months 3–6 podcasts emphasize overcoming barriers, focusing on nutrition and exercise. | - Interest and trust; Media consumption; Evaluation of the media; Intention; Knowledge questions |
| Wilcox 2022, USA  Wilcox 2018  Wilcox 2022 | RCT, 6m | n=219 pregnant women.    Intervention:  Female: 100%  30.4±5.2 y    Standard care:  Female: 100%  29.1±4.8 y | Analyse how a behavioural intervention affects PA, sedentary behaviour, steps, dietary habits, and HRQOL. | Behavioural podcasts - Weight gain in pregnancy  Healthy eating  PA  Diabetes Prevention Program sessions and focused on gradual weight loss | Both groups receive counselling sessions, participate in a Facebook group, and have access to 10 podcasts. The podcasts are cantered around promoting a healthy pregnancy, focusing on behavioural strategies, PA, and diet topics. | - PA - Dietary intake - Health related QoL |

Supplementary content 4. The Mixed Methods Appraisal Tool (MMAT) scores

|  | **Screening questions** | | **Qualitative studies** | | | | |
| --- | --- | --- | --- | --- | --- | --- | --- |
| **Study** | **1** | **2** | **1.1** | **1.2** | **1.3** | **1.4** | **1.5** |
| Edwards (2021) | Y | Y | Y | Y | Y | Y | Y |
| Knudsen (2022) | Y | Y | Y | Y | Y | Y | Y |
| Perry (2022) | Y | Y | Y | Y | Y | Y | Y |
| Semakula (2019) | Y | Y | Y | Y | Y | Y | Y |
| Shaw (2022) | Y | Y | Y | Y | Y | Y | Y |
| Y: Yes.  1: Are there clear research questions? 2; Do the collected data allow to address the research questions?  1.1. Is the qualitative approach appropriate to answer the research question? 1.2. Are the qualitative data collection methods adequate to address the research question? 1.3. Are the findings adequately derived from the data? 1.4. Is the interpretation of results sufficiently substantiated by data? 1.5. Is there coherence between qualitative data sources, collection, analysis and interpretation? | | | | | | | |

|  | **Screening questions** | | **Randomised controlled trials** | | | | | **Non-randomised trials** | | | | | **Quantitative descriptive studies** | | | | | **Mixed methods studies** | | | | |
| --- | --- | --- | --- | --- | --- | --- | --- | --- | --- | --- | --- | --- | --- | --- | --- | --- | --- | --- | --- | --- | --- | --- |
| **Study** | **1** | **2** | **2.1** | **2.2** | **2.3** | **2.4** | **2.5** | **3.1** | **3.2** | **3.3** | **3.4** | **3.5** | **4.1** | **4.2** | **4.3** | **4.4** | **4.5** | **5.1** | **5.2** | **5.3** | **5.4** | **5.5** |
| Balls-Berry (2018) | Y | Y |  |  |  |  |  |  |  |  |  |  | U | U | Y | U | Y |  |  |  |  |  |
| Bangia (2014) | Y | Y |  |  |  |  |  |  |  |  |  |  |  |  |  |  |  | Y | Y | Y | Y | Y |
| Bangia (2017) | Y | Y |  |  |  |  |  | Y | Y | Y | N | Y |  |  |  |  |  |  |  |  |  |  |
| Biber (2021) | Y | Y |  |  |  |  |  | Y | Y | Y | Y | Y |  |  |  |  |  |  |  |  |  |  |
| Cai (2023) | Y | Y | U | Y | Y | N | Y |  |  |  |  |  |  |  |  |  |  |  |  |  |  |  |
| Caoilte (2023) | Y | Y |  |  |  |  |  |  |  |  |  |  | Y | Y | Y | N/A | Y |  |  |  |  |  |
| Carrotte (2023) | Y | Y |  |  |  |  |  |  |  |  |  |  | Y | Y | Y | N/A | Y |  |  |  |  |  |
| Conde-Ferráez (2022) | Y | Y |  |  |  |  |  |  |  |  |  |  |  |  |  |  |  | Y | Y | U | N | U |
| Dreer (2021) | Y | Y |  |  |  |  |  | Y | Y | Y | U | Y |  |  |  |  |  |  |  |  |  |  |
| Dunn (2019) | Y | Y |  |  |  |  |  |  |  |  |  |  | Y | Y | Y | U | Y |  |  |  |  |  |
| Fine (2022) | Y | Y |  |  |  |  |  |  |  |  |  |  | Y | N/A | Y | N/A | Y |  |  |  |  |  |
| Goodridge (2021) | Y | Y |  |  |  |  |  |  |  |  |  |  |  |  |  |  |  | Y | Y | Y | Y | Y |
| Hales (2016a) | Y | Y |  |  |  |  |  |  |  |  |  |  |  |  |  |  |  | Y | Y | Y | Y | Y |
| Hales (2017) | Y | Y | U | U | Y | U | Y |  |  |  |  |  |  |  |  |  |  |  |  |  |  |  |
| Hales (2016b) | Y | Y | Y | N | Y | N | Y |  |  |  |  |  |  |  |  |  |  |  |  |  |  |  |
| Huberty (2020) | Y | Y |  |  |  |  |  | Y | Y | Y | Y | Y |  |  |  |  |  |  |  |  |  |  |
| Johnson (2012) | Y | Y |  |  |  |  |  | Y | Y | Y | Y | Y |  |  |  |  |  |  |  |  |  |  |
| Karing (2022) | Y | Y | U | Y | Y | U | Y |  |  |  |  |  |  |  |  |  |  |  |  |  |  |  |
| Kirkpatrick (2021) | Y | Y |  |  |  |  |  |  |  |  |  |  | Y | Y | Y | Y | Y |  |  |  |  |  |
| Ko (2014) | Y | Y | U | Y | Y | U | Y |  |  |  |  |  |  |  |  |  |  |  |  |  |  |  |
| LaBrosse (2013) | Y | Y |  |  |  |  |  | Y | Y | Y | Y | Y |  |  |  |  |  |  |  |  |  |  |
| Laird (2022) | Y | Y | Y | U | N | U | Y |  |  |  |  |  |  |  |  |  |  |  |  |  |  |  |
| Lauricella (2014) | Y | Y |  |  |  |  |  |  |  |  |  |  |  |  |  |  |  | Y | Y | Y | Y | Y |
| Leite (2022) | Y | Y |  |  |  |  |  |  |  |  |  |  |  |  |  |  |  | Y | Y | Y | Y | Y |
| LeRouge (2014) | Y | Y |  |  |  |  |  |  |  |  |  |  | Y | Y | Y | N/A | Y |  |  |  |  |  |
| Mailey (2016) | Y | Y |  |  |  |  |  |  |  |  |  |  |  |  |  |  |  | Y | Y | Y | Y | Y |
| Mailey at al (2019) | Y | Y |  |  |  |  |  |  |  |  |  |  |  |  |  |  |  | Y | Y | Y | Y | Y |
| McCall (2022) | Y | Y |  |  |  |  |  |  |  |  |  |  | Y | Y | Y | N/A | Y |  |  |  |  |  |
| Militello (2021) | Y | Y |  |  |  |  |  |  |  |  |  |  |  |  |  |  |  | Y | Y | Y | Y | Y |
| Mugisha (2022) | Y | Y |  |  |  |  |  |  |  |  |  |  | Y | Y | Y | U | U |  |  |  |  |  |
| Semakula (2020) | Y | Y |  |  |  |  |  |  |  |  |  |  |  |  |  |  |  | Y | N | N | Y | Y |
| Semakula (2017) | Y | Y | Y | Y | Y | N | Y |  |  |  |  |  |  |  |  |  |  |  |  |  |  |  |
| Turner-McGrievy  (2009) | Y | Y | U | Y | Y | U | Y |  |  |  |  |  |  |  |  |  |  |  |  |  |  |  |
| Turner-McGrievy (2013a) | Y | Y |  |  |  |  |  |  |  |  |  |  | Y | Y | Y | Y | Y |  |  |  |  |  |
| Turner-McGrievy  (2013b) | Y | Y |  |  |  |  |  |  |  |  |  |  | Y | Y | Y | N/A | Y |  |  |  |  |  |
| Turner-McGrievy  (2017) | Y | Y |  |  |  |  |  |  |  |  |  |  | Y | Y | Y | Y | Y |  |  |  |  |  |
| Turner-McGrievy  (2020) | Y | Y |  |  |  |  |  |  |  |  |  |  | Y | Y | Y | N/A | Y |  |  |  |  |  |
| Turner-McGrievy  (2019) | Y | Y |  |  |  |  |  |  |  |  |  |  | Y | Y | Y | N/A | Y |  |  |  |  |  |
| Turner-McGrievy  (2014) |  |  |  |  |  |  |  |  |  |  |  |  | Y | Y | Y | N/A | Y |  |  |  |  |  |
| Turner-McGrievy  (2017b) | Y | Y | Y | Y | Y | Y | Y |  |  |  |  |  |  |  |  |  |  |  |  |  |  |  |
| Turner-McGrievy (2011) | Y | Y |  |  |  |  |  |  |  |  |  |  | Y | Y | Y | N | Y |  |  |  |  |  |
| Weib (2023) | Y | Y | N | N | Y | N | Y |  |  |  |  |  |  |  |  |  |  |  |  |  |  |  |
| Wilcox (2022) | Y | Y | N/A | N/A | N/A | N/A | N/A |  |  |  |  |  |  |  |  |  |  |  |  |  |  |  |
| Wilcox (2018) | Y | Y | Y | U | N/A | Y | N/A |  |  |  |  |  |  |  |  |  |  |  |  |  |  |  |
| Wilcox (2022) | Y | Y | Y | Y | Y | Y |  |  |  |  |  |  |  |  |  |  |  |  |  |  |  |  |
| Y: Yes; N: No; U: Unclear.  **Randomised controlled trials:** 2.1. Is randomization appropriately performed? 2.2. Are the groups comparable at baseline? 2.3. Are there complete outcome data? 2.4. Are outcome assessors blinded to the intervention provided? 2.5 Did the participants adhere to the assigned intervention?  **Non-randomised trials:** 3.1. Are the participants representative of the target population? 3.2. Are measurements appropriate regarding both the outcome and intervention (or exposure)? 3.3. Are there complete outcome data?  3.4. Are the confounders accounted for in the design and analysis? 3.5. During the study period, is the intervention administered (or exposure occurred) as intended?  **Quantitative descriptive studies:** 4.1. Is the sampling strategy relevant to address the research question? 4.2. Is the sample representative of the target population? 4.3. Are the measurements appropriate? 4.4. Is the risk of nonresponse bias low? 4.5. Is the statistical analysis appropriate to answer the research question?  **Mixed methods studies:** 5.1. Is there an adequate rationale for using a mixed methods design to address the research question? 5.2. Are the different components of the study effectively integrated to answer the research question? 5.3. Are the outputs of the integration of qualitative and quantitative components adequately interpreted? 5.4. Are divergences and inconsistencies between quantitative and qualitative results adequately addressed? 5.5. Do the different components of the study adhere to the quality criteria of each tradition of the methods involved? | | | | | | | | | | | | | | | | | | | | | | |
